# Peripheral Blood Biomarkers Associated With Improved Functional Outcome in Patients With Chronic Left Ventricular Dysfunction: A Biorepository Evaluation of the FOCUS-CCTRN Trial

**DOI:** 10.3389/fcvm.2021.698088

**Published:** 2021-09-03

**Authors:** Lourdes Chacon Alberty, Emerson C. Perin, James T. Willerson, Amir Gahremanpour, Roberto Bolli, Phillip C. Yang, Jay H. Traverse, Dejian Lai, Carl J. Pepine, Doris A. Taylor

**Affiliations:** ^1^Regenerative Medicine Department, Texas Heart Institute, Houston, TX, United States; ^2^Stem Cell Center, Texas Heart Institute, Houston, TX, United States; ^3^Hospital Corporation of America-Houston Heart, Houston, TX, United States; ^4^School of Medicine, University of Louisville, Louisville, KY, United States; ^5^Stanford University School of Medicine, Stanford, CA, United States; ^6^Minneapolis Heart Institute Foundation at Abbott Northwestern Hospital and University of Minnesota School of Medicine, Minneapolis, MN, United States; ^7^UTHealth School of Public Health, Houston, TX, United States; ^8^University of Florida College of Medicine, Gainesville, FL, United States

**Keywords:** cell therapy, heart failure, stem cells, ventricular dysfunction, B-lymphocytes, immune system, heart, B-Cells

## Abstract

Cell therapy trials for heart failure (HF) have shown modest improvement; however, the mechanisms underlying improvement in some patients but not others are not well understood. Although immune cells are important in the course of HF, our understanding of the immune processes in HF is limited. The objective of this study was to evaluate associations between temporal changes in peripheral blood (PB) cell subpopulations and improved outcome in patients with chronic ischemic cardiomyopathy after bone marrow-derived mononuclear cell therapy or placebo in the FOCUS-CCTRN trial. Peripheral blood was collected at days 0, 1, 30, 90, and 180 from consented participants. We used flow cytometry to compare PB populations in patients with the best (cohort 1) or worst functional outcome (cohort 2) in three primary endpoints: left ventricular (LV) ejection fraction, LV end-systolic volume, and maximal oxygen consumption (VO_2_ max). A linear mixed model was used to assess changes over time in 32 cell populations. The difference between each time point and baseline was calculated as linear contrast. Compared with cohort 2, patients who improved (cohort 1) had a higher frequency of CD45^+^CD19^+^ B cells at days 0, 1, 90, and 180. CD11B^+^ cells increased over baseline at day 1 in both cohorts and remained higher in cohort 2 until day 30. CD45^+^CD133^+^ progenitor cells decreased over baseline at day 30 in cohort 1. We identified specific cell subpopulations associated with improved cardiac function in patients with chronic LV dysfunction. These findings may improve patient selection and prediction of outcomes in cell therapy trials.

## Introduction

Heart failure (HF) affects more than 38 million patients globally and poses enormous challenges for health care systems worldwide (1, 2). In the United States, a 46% increase in the prevalence of HF and an almost 127% increase in total medical costs are predicted by 2030 (3). Despite progress over the past three decades, the prognosis of patients with HF remains poor, and current therapies do not address the underlying conditions. Therefore, understanding the mechanisms of HF is critical for developing more effective therapies (4).

The Cardiovascular Cell Therapy Research Network (CCTRN) completed the Effectiveness of Stem Cell Treatment for Adults with Ischemic Cardiomyopathy (FOCUS-CCTRN) trial, a randomized controlled study comparing therapy with autologous bone marrow mononuclear cells (BM-MNCs; 100 × 10^6^ cells) with placebo in patients with ischemic HF. The trial showed a significant improvement in left ventricular ejection fraction (LVEF) (2.7% [95% CI, 0.3 to 5.1]; *p* = 0.03) between cell and placebo-treated patients at 6 months (5). Moreover, in a meta-analysis of clinical cell therapy in HF, cell delivery was associated with improved quality of life and New York Heart Association (NYHA) classification and decreased length of hospital stay, re-hospitalization, and incidence of HF (6). Furthermore, we previously reported that a subset of patients in the FOCUS-CCTRN trial showed improved LVEF, maximal oxygen consumption (VO_2_ max), and left ventricular end-systolic volume (LVESV). In this patient subgroup, improvement in the three outcomes was associated with an increase in bone marrow CD19^+^ B cells, CD11b^+^ monocytes, CD31^dim^ cell subsets, and CXCR4^+^ migratory cells and a reduction in CD31^bright^ cells compared with patients who had no change or a worsening in any of the three outcomes (7, 8). However, it is not known if this cell population profile persists in the peripheral blood (PB).

Heart failure is a systemic syndrome with many etiologies, frequently driven by myocardial dysfunction. Over the last decade, the role of the immune system in cardiovascular conditions has become more apparent, generating interest in understanding the role of circulating immune and reparative cells in the pathogenesis of cardiac disease, and the possibility of discovering targeted new therapies. Although the development and progression of HF have been traditionally characterized as a hemodynamic disorder, mounting evidence suggests that immune cells may be an important contributor to this process (9, 10). Given the promising impact of cell therapy in HF (11, 12) and the previous reports by us and others on the association of specific immune and progenitor cells with improved outcome (7, 13, 14), we sought to characterize the associations between baseline characteristics, temporal changes in circulating immune and progenitor cells, and clinical outcomes in FOCUS-CCTRN patients. Our objective is to identify the biological variables that may help in selecting patients who are more likely to respond positively to cell therapy. These findings could be used to select patients for inclusion in clinical trials and to choose appropriate cell products for treating individual patients, eventually resulting in more targeted precision therapy.

## Methods

### Study Design

**Figure 1:**
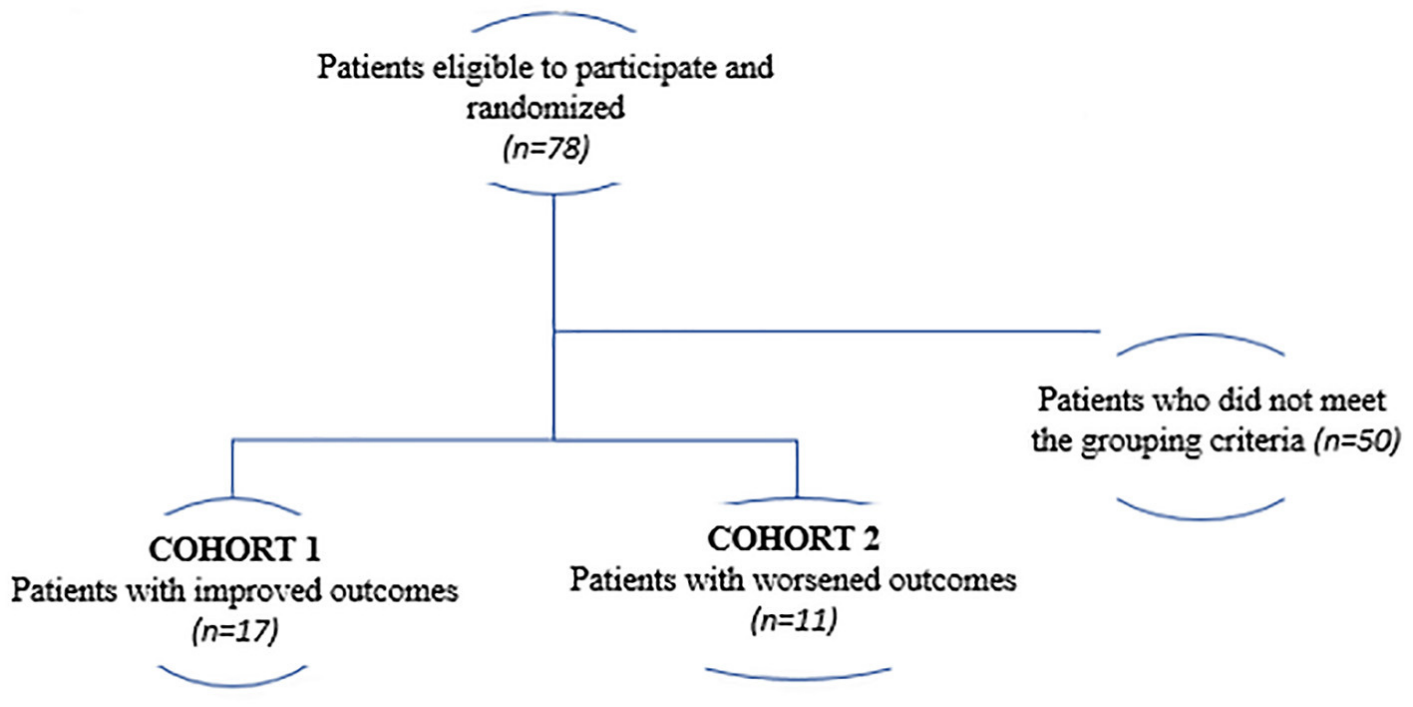
Flowchart showing the grouping strategy used in this study.

The present study is a secondary analysis from the FOCUS-CCTRN trial (5). Here, we sought to investigate whether changes in PB-derived circulatory cell subpopulations collected at different time-points and demographic parameters measured at baseline could predict improved LVEF, VO_2_ max, and LVESV in HF patients regardless of treatment. Patients were classified as having either the best functional outcomes (cohort 1; *n* = 17) or the worst outcomes (cohort 2; *n* = 11). The best functional outcome was defined as an increase in LVEF and VO_2_ max and a decrease in LVESV, whereas the worst outcome was defined as a decrease in LVEF and VO_2_ max, and an increase in LVESV (Figure 1).

FOCUS-CCTRN was a National Institutes of Health (NIH)-sponsored multicenter, randomized, double-blind, placebo-controlled trial that evaluated transendocardial injections of either 100 × 10^6^ autologous BM-MNCs or placebo (cell-free) preparation (5, 15, 16) in patients with ischemic HF and reduced LVEF (<45%). Left ventricular end-systolic volume was assessed by using echocardiography adjudicated by an independent echocardiography core laboratory and was calculated by using a modified biplane Simpson method with myocardial contrast. VO_2_ max was assessed by the Naughton treadmill protocol. Adenosine myocardial perfusion (SPECT) test was performed to identify changes in ischemic (reversible) defects from rest and after adenosine infusion over 4 min. The trial was conducted at five CCTRN sites and featured a data coordinating center, a protocol review committee, and a data safety and monitoring board appointed by the NIH. All patients provided written informed consent, and the institutional review board at each study center approved the protocol, including biospecimen collection and analyses. The biorepository protocol was approved by the committee for the protection of human subjects at the University of Texas Health Science Center at Houston.

### Study Procedures

Peripheral blood samples were collected on day 0 (before administration of the cell product or placebo) and on days 1, 30, 90, and 180 after treatment. We collected 20 ml of PB by venipuncture into vacutainers (BD Biosciences, Bedford, MA) containing ethylenediaminetetraacetic acid. The samples were shipped immediately at 4°C to the CCTRN Biorepository Core laboratory. Red blood cells were lysed with ammonium chloride, and the remaining cells were washed twice with phosphate-buffered saline (PBS) and resuspended in fluorescence-activated cell sorting (FACS) buffer containing PBS and 2.5% fetal bovine serum. The concentration and viability of the nucleated cells were determined with an automated cell counter (Guava Via Count, EMD Millipore Corp., Billerica, MA). Approximately 1 × 10^6^ to 5 × 10^6^ cells were stained with antibodies against human CD45, CD34, CD31, CD133, vascular endothelial growth factor receptor-2, chemokine (C-X-C motif) receptor 4 (CXCR4), CD3, CD11, CD14, and CD19, as well as their corresponding isotype controls (Table 1). Cells were incubated with the antibodies for 20 min at 4°C in the dark. Then, the cells were washed twice with PBS and suspended in FACS buffer.

**Table 1:** Cell surface marker expression used to report phenotypes of peripheral blood-derived mononuclear cells in the FOCUS-CCTRN trial (7).

**Single-positive cell surface marker profiles**	**Double-positive cell surface marker profiles**	**Triple-positive cell surface marker profiles**
CD3^+^ CD31^dim^ KDR^+^ CD133^+^ CD14^+^ CD11b^+^ CD45^+^ CD31^+^ CXCR4^+^ CD19^+^	CD45^+^CD31^bright^ CD31^+^CD34^−^ CD45^+^CD11b^+^ CD45^+^CXCR4^−^ CD45^+^CD3^+^ CD45^+^CXCR4^dim^ CD45^+^CD14^−^ CD45^+^CD11b^−^ CD34^+^CD133^+^ CD45^+^CD133^+^ CD45^+^CD14^+^ CD34^+^CD31^+^ CD34^+^KDR^+^ CD45brightCXCR4^+^ CD45^+^CD11b^−^ CD45^+^CD31^+^ CD45^+^CD31^dim^ CD45^+^CXCR4^interm^ CD19^+^CD11b^+^ CD45^+^CD19^+^ CD19^+^CXCR4^+^	CD133^+^CD34^+^KDR^+^

**Figure 2:**
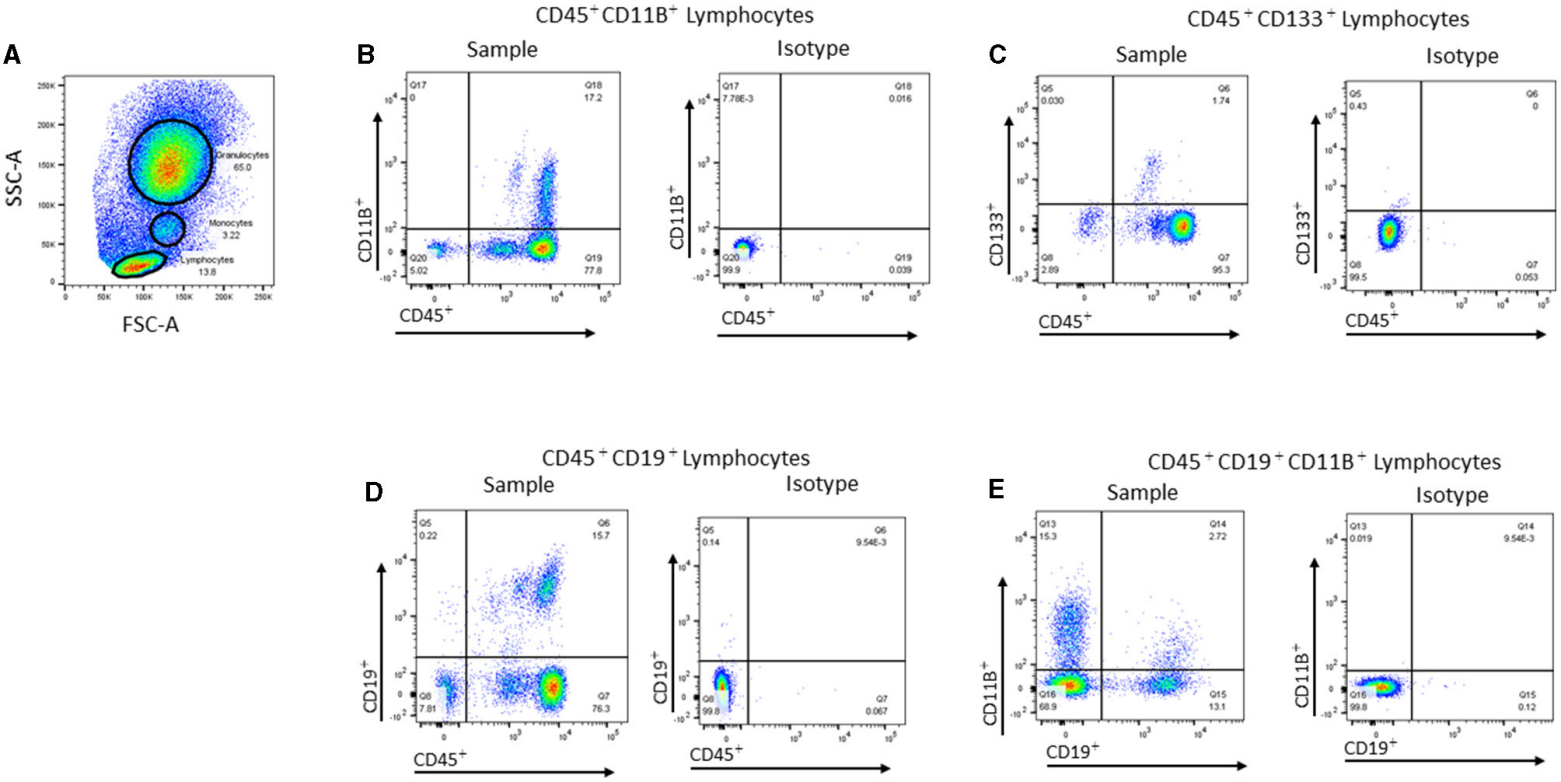
Representative flow cytometric gating strategies used for analyzing peripheral blood cell populations. **(A)** Representative dot plot showing the gates used to identify lymphocytes, monocytes, and granulocytes in the peripheral blood (PB) based on forward scatter (FSC-A) vs. side scatter (SSC-A). **(B)** Representative dot plot showing CD45^+^CD11B^+^ cells within the monocyte gate and the corresponding isotype control. **(C)** Representative dot plot showing CD45^+^CD133^+^ cells within the lymphocyte gate and the corresponding isotype. **(D)** Representative dot plot showing CD45^+^CD19^+^ cells within the lymphocyte gate and the corresponding isotype. **(E)** Representative dot plot showing CD19^+^CD11B^+^ within the lymphocyte gate and the corresponding isotype.

Multiparameter polychromatic analyses were performed by using an LSR II flow cytometer (Becton, Dickinson and Company, Franklin Lakes, NJ) with Diva software. The flow cytometry data were analyzed with FlowJo software (Tree Star Inc., Ashland, OR) by using standardized guidelines for cell gating (Figure 2). The extracellular markers were assessed individually or in combination to determine the frequencies of 32 cell populations (Table 1), including immune, hematopoietic, and stem/progenitor cells (15).

### Endpoints

As described previously (7, 8), we performed an embedded cohort study of 78 of the 92 randomized patients from the FOCUS-CCTRN trial who had complete follow-up data and who consented to have their PB analyzed. We used a rigorous criterion of best functional outcome, which required the following improvements: increased LVEF, increased VO_2_ max, and decreased LVESV. Our goal was to identify associations between the circulating cell profile and cardiac functional outcomes.

### Statistical Analyses

Patient clinical and demographic variables were compared using a Student *t*-test for continuous variables, and the data are expressed as mean ± SD or quartile. The Wilcoxon two-sample test was used to evaluate non-normally distributed data. The Fisher's exact test was used to compare dichotomous variables. Differences in NYHA classification and body mass index were assessed by the chi-square test.

The frequency of PB mononuclear cell populations was measured at days 0 (baseline), 1, 30, 90, and 180 after injections. We evaluated changes in cell frequencies over time within cohorts. For each cell phenotype, a linear mixed model (STATA mixed module with unstructured residual matrix) was used to determine the changes over time in the frequencies of each cell population. We entered the following factors: time after infusion of cells or placebo (as a categorical variable with five values), diabetes, smoking status, baseline age (categorical variable as older/younger than 60 years), and sex. The difference between each study timepoint to baseline was calculated as a linear contrast.

Multiple logistic regression analysis was performed to further determine association between changes in endpoints (LVEF, LVESV, and VO_2_ max) from baseline to 6 months and changes from baseline in the frequency of PB circulating cells. The model was adjusted by common cardiac risk factors: age, smoking history, and diabetes. Statistical analyses were conducted using STATA (Stata Corp LLC, College Station, Texas, USA), and a two-sided significance testing was used. *P*-value <0.05 was considered significant. Because of the exploratory nature of this study, *p*-values were not adjusted for multiple comparisons.

## Results

### Baseline Characteristics and Clinical Outcomes

**Figure 3:**
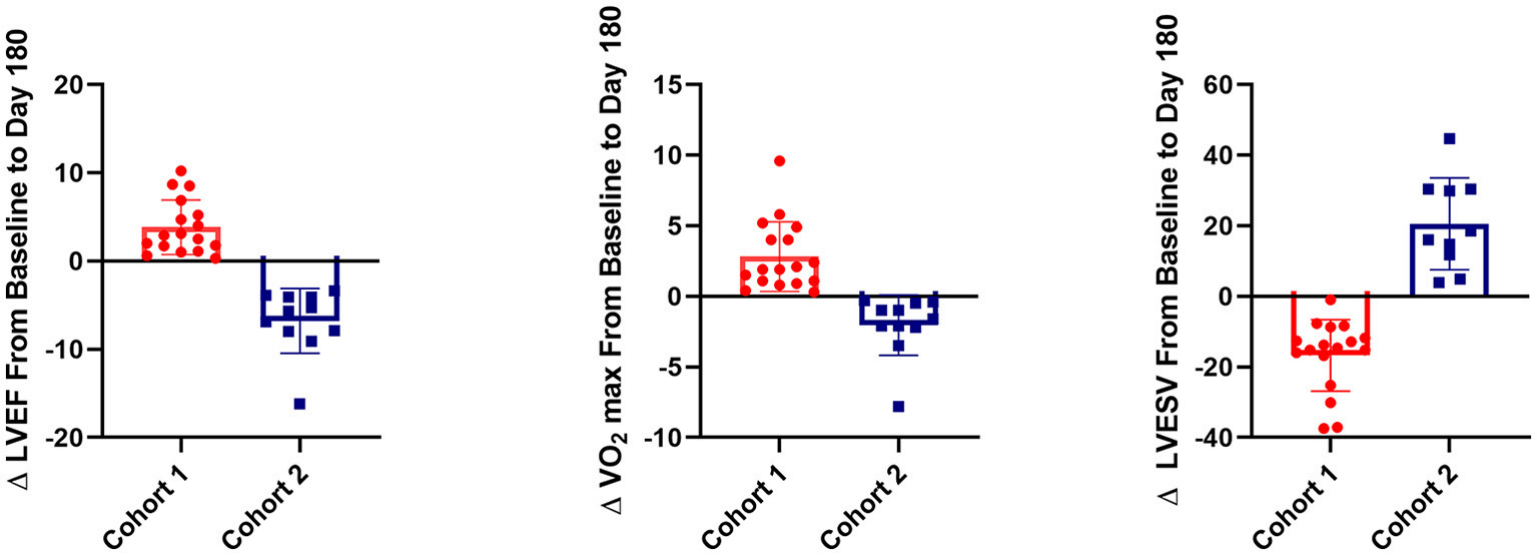
3D plot of change in left ventricular ejection fraction (LVEF), left ventricular end-systolic volume (LVESV), and VO_2_ max between baseline and post-operative day 180 in the two cohorts from the FOCUS-CCTRN trial. Cohort 1 showed an increase in LVEF (*p* < 0.001) and VO_2_ max (*p* < 0.001) and a decrease in LVESV (*p* < 0.001) when compared with cohort 2. Percentage of CD45^+^CD19^+^ cells at baseline is represented by the size of circles. Larger circles indicate a higher percentage of CD45^+^CD19^+^ cells, and smaller circles indicate a lower percentage.

We compared baseline characteristics and outcomes in patients who had the best functional outcome (cohort 1) with those who had the worst functional outcome (cohort 2). Cohort 1 showed an increase in LVEF of 3.75 ± 2.95% and in VO_2_ max of 2.82 ± 2.47 ml/kg/min and a decrease in LVESV of −16.48 ± 10.20 ml. In contrast, cohort 2 demonstrated a decrease in LVEF of −6.78 ± 3.66% and in VO_2_ max of −2.045 ± 2.14 ml/kg/min and an increase in LVESV of 26.13 ± 22.28 ml (Figure 3). At baseline (Table 2), cohort 1 had lower brain natriuretic peptide (BNP) levels (68.5 pg/ml [51.0–128.1] vs. 256 pg/ml [143–448.4]; *p* = 0.029, respectively). Cohort 2 had more patients in NYHA class III (i.e., with more severe HF symptoms) than did cohort 1 (63.64 vs. 23.53%; *p* = 0.03, respectively) and were older than cohort 1 (69.53 ± 9.04 vs. 60.37 ± 11.98 years; *p* = 0.040, respectively). We did not observe differences at baseline in regional wall motion in the echocardiographic measurements, but interestingly, there was a decreased delta in wall motion in cohort 1. In the SPECT test, the cohorts did not differ in the percentage of reversible defect, total myocardial reversible defect, or total myocardial defect. We did not find any differences in the history of smoking, diabetes, or hypertension between the cohorts (Table 2).

**Table 2:** Clinical characteristics of the study cohorts.

**Variables**	**Cohort 1 (***n*** = 17)**	**Cohort 2 (***n*** = 11)**	***p***-**value**
Age in years, mean (SD)	60.37 (11.98)	69.53 (9.04)	0.040
Female, *n* (%)	3 (17.65%)	2 (18.18%)	1.00
Caucasian, *n* (%)	17 (100%)	11 (100%)	1.00
Glomerular filtration rate at baseline, mean (SD)	82.74 (31.99)	66.86 (13.66)	0.133
Hemoglobin at baseline (gm/dl), mean (SD)	13.94 (2.04)	14.40 (0.91)	0.490
Body mass index at baseline (kg/m^2^) mean (SD)	30.25 (5.47)	30.02 (7.29)	0.817
<25, *n* (%)	3 (17.65%)	2 (18.18%)	1.00
>25–30, *n* (%)	6 (35.29%)	4 (36.36%)	
>30, *n* (%)	8 (47.06%)	5 (45.45%)	
**Cardiac function at baseline**			
LVEF (%), mean (SD)	35.3 (10.90)	35.8 (8.7)	0.862
End diastolic volume (ml), mean (SD)	174.81 (55.15)	184.25 (58.78)	0.547
End systolic volume (ml), mean (SD)	116.36 (53.18)	120.63 (46.21)	0.829
VO_2_ max (ml/kg/min), mean (SD)	15.11 (4.05)	15.64 (4.85)	0.759
Echo wall motion, mean (SD)	3.53 (0.70)	3.43(0.52)	0.690
Percentage of reversible defect	22.44 (25.75)	16.55 (26.95)	0.264
Percentage total myocardial reversible defect, mean (SD)	6.63 (9.19)	7.27 (12.12)	0.346
Total myocardial defect, mean (SD)	31.13 (8.91)	34.73 (10.37)	0.343
**Cardiac function at day 180**			
LVEF (%), mean (SD)	39.05 (12.90)	29.00 (6.95)	0.049
End diastolic volume (ml), mean (SD)	159.27 (51.42)	203.15 (59.83)	0.233
End systolic volume (ml), mean (SD)	100.13 (50.81)	133.83 (51.97)	0.116
VO_2_ max (ml/kg/min), mean (SD)	17.93 (5.14)	13.59 (3.92)	0.025
Echo wall motion	3.39 (0.81)	3.86 (0.59)	0.107
Percentage of reversible defect	17.64 (27.07)	23.13 (27.65)	0.517
Percentage total myocardial reversible defect, mean (SD)	6.28 (6.32)	6.45 (11.97)	0.457
Total myocardial defect, mean (SD)	31.94(10.91)	35.09 (7.39)	0.378
Δ Echo wall motion, mean (SD)	−0.16(0.33)	0.43 (0.56)	0.002
Δ Percentage of reversible defect, mean (SD)	0.67 (21.82)	1.69 (24.82)	0.966
Δ Percentage of total myocardial reversible defect, mean (SD)	0.69 (24.82)	1.091 (22.35)	0.990
Δ total myocardial defect, mean (SD)	0.81 (5.50)	0.36 (11.48)	0.652
BNP at baseline (pg/ml)*	68.5 (51.0–128.1)	258 (143–448.4)	0.029
BNP at day 180 (pg/ml)*	73.5 (54–130)	250 (124.5–379)	0.060
**NYHA class at baseline**			0.070
Grade I baseline, *n* (%)	3 (17.65%)	2 (18.18%)	
Grade II baseline, *n* (%)	10 (58.82%)	2 (18.18 %)	
Grade III baseline, *n* (%)	4 (23.53%)	7 (63.64%)	
Grade IV baseline, *n* (%)	0 (0%)	0 (0%)	
**NYHA class at day 180^†^**			0.239
Grade I, *n* (%)	8 (47.06%)	2 (18.18%)	
Grade II, *n* (%)	5 (29.41%)	6 (54.55%)	
Grade III, *n* (%)	3 (17.65%)	3 (27.27%)	
Grade IV, *n* (%)	0 (0%)	0 (0%)	
Distance walked baseline (ft.), mean (SD)	1120.94 (363.81)	891.68(494.715)	0.169
Distance walked at day 180 (ft.), mean (SD)	1258.706 (334.59)	971.66 (538.64)	0.112
Δ Distance walked (ft.), mean (SD)	−154.43 (407.25)	−26.21 (511.26)	0.497
**Medications**			
Aspirin, *n* (%)	13 (76.47%)	10 (90.91%)	0.619
Betablockers, *n* (%)	16 (94.12%)	11 (100%)	1.00
Calcium channel blockers, *n* (%)	1 (5.88%)	1 (9.09%)	1.00
Cholesterol lowering, *n* (%)	15 (88.24%)	9 (81.81%)	1.00
Digitalis, *n* (%)	1 (5.88%)	2 (18.18%)	0.34
Diuretics, *n* (%)	11 (64.71%)	7 (63.63 %)	1.00
Insulin, *n* (%)	1 (5.88%)	3 (27.27%)	0.269
Nitrates, *n* (%)	8 (47.06%)	8 (72.73%)	0.253
Pain medication, *n* (%)	4 (23.53%)	2 (18.18%)	1.00
Ranexa, *n* (%)	4 (23.53%)	3 (27.27%)	1.00
Statins, *n* (%)	15 (88.24%)	7 (63.64 %)	0.092
Antiarrhythmics, *n* (%)	3 (17.65%)	2 (18.18%)	1.00
BMMNC therapy, *n* (%)	16 (94.12%)	7 (63.64%)	0.062
**Medical history**			
Angina, *n* (%)	5 (29.41%)	3 (27.27%)	1.00
Smoking history, *n* (%)	13 (76.47%)	9 (81.18%)	1.00
Diabetes, *n* (%)	5 (29.41%)	3 (27.27%)	1.00
Hypertension, *n* (%)	14 (82.35%)	10(90.91%)	1.00
CHF hospitalization, *n* (%)	5 (29.41%)	3 (27.27%)	1.00
History of arrhythmias, *n* (%)	10 (58.82%)	5 (45.45%)	0.706
History of CABG, *n* (%)	7 (41.18%)	9 (81.81%)	0.054
History of CHF, *n* (%)	10 (58.82%)	7 (63.64%)	1.00
History of AMI, *n* (%)	15 (88.24%)	11 (100 %)	0.505
History of PVD, *n* (%)	3 (17.65%)	2 (18.18%)	1.00
History of stroke, *n* (%)	0 (0%)	2 (18.18%)	0.146
History of TIA, *n* (%)	1 (5.88%)	2 (18.18%)	0.543
History of VHD, *n* (%)	6 (35.29%)	2 (18.18%)	0.419
Hyperlipidemia, *n* (%)	17 (100%)	10 (90.91%)	0.393
Prior pacemaker, *n* (%)	12 (70.59%)	10 (90.91%)	0.355
Prior revascularizations, *n* (%)	15 (88.24%)	9 (81.81%)	1.00

*AMI, acute myocardial infraction; BMMNC, bone marrow mononuclear cell; BNP, B-type natriuretic peptide; CABG, coronary artery bypass graft; CHF, congestive heart failure; LVEF, left ventricular ejection fraction; NYHA, New York Heart Association; PVD, peripheral vascular disease; TIA, transient ischemic attack; VHD, valvular heart disease; VO_2_ max, maximal oxygen consumption. The data are presented as percentages or numbers or mean ± SD, unless otherwise specified*.

* *Results reported as median (first quartile – third quartile), cohort 1 = 10; cohort 2 = 11*.

† *NYHA class was not recorded for one of the patients in cohort 1*.

### Differences in Circulating Cell Populations

**Figure 4:**
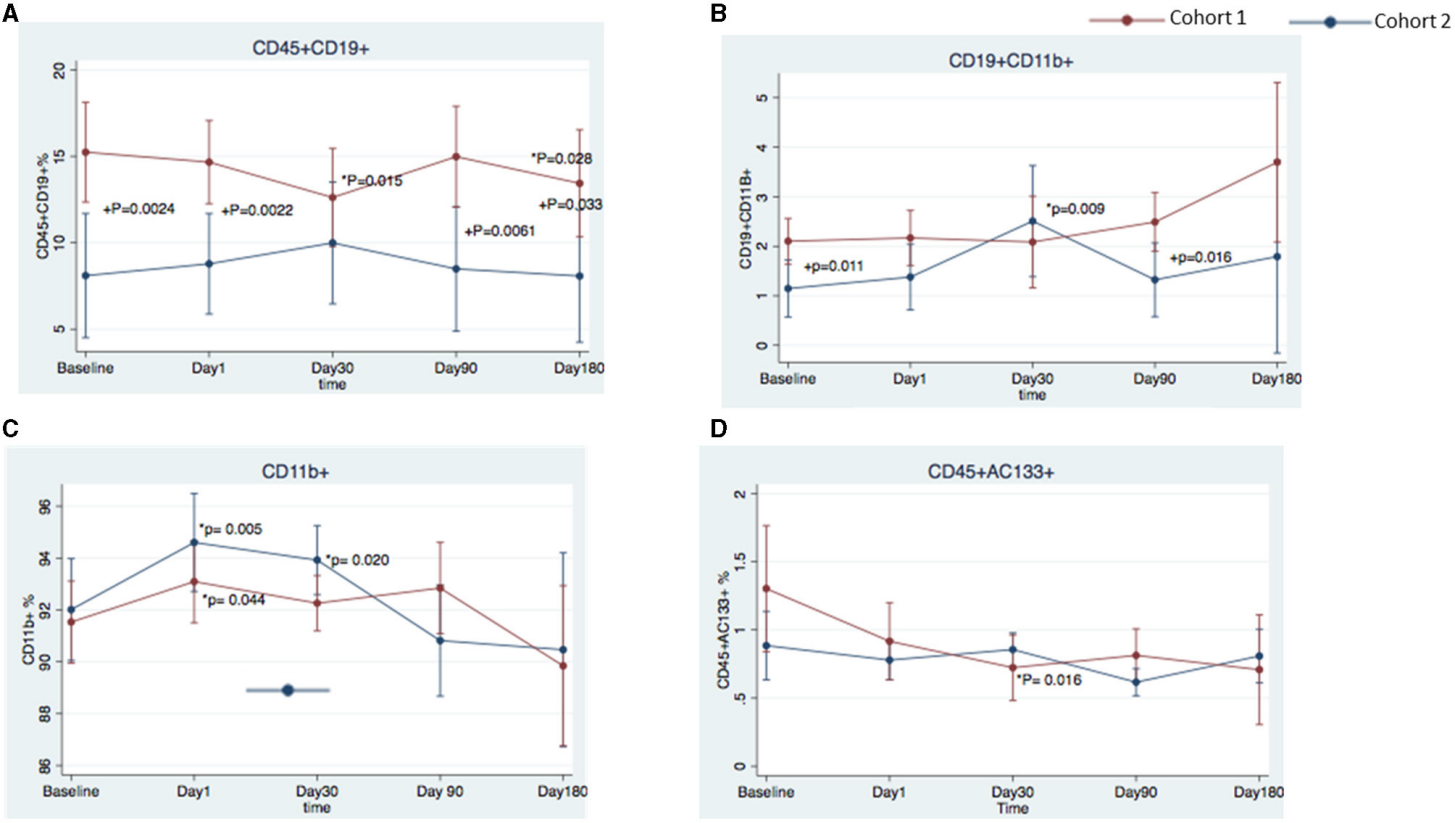
Temporal changes in peripheral blood cell populations in cohort 1 and cohort 2 from the FOCUS-CCTRN trial. Temporal changes in peripheral blood CD45^+^CD19^+^ cells **(A)**, CD19^+^ CD11B^+^ cells **(B)**, CD11B^+^ cells **(C)**, and CD45^+^CD133^+^ cells **(D)**. *P = changes over time vs. baseline, ^+^*P* = cohort 1 vs. cohort 2.

Patients with the best functional outcome (cohort 1) had a higher frequency of CD45^+^CD19^+^ B cells at all timepoints than did patients in the worst outcome group (cohort 2) (baseline, 15.24 ± 7.55 vs. 8.95 ± 3.49%, respectively, *p* = 0.0024; day 1, 14.67 ± 5.53 vs. 8.78 ± 4.51%, *p* = 0.0022; day 90, 14.98 ± 6.64 vs. 8.49 ± 4.33%, *p* = 0.0061; and day 180, 13.44 ± 7.85 vs. 8.07 ± 4.34%, *p* = 0.033) (Figures 3, 4A). Cohort 1 had a higher percentage of the CD19^+^CD11B^+^ B cell subset at baseline (2.10 ± 1.20 vs. 1.15 ± 0.58%, *p* = 0.011) and day 90 (2.49 ± 1.50 vs. 1.32 ± 0.62%, *p* = 0.016), whereas cohort 2 had an increase in this B cell subset at day 30 when compared to baseline (1.15 ± 0.29 vs. 2.51 ± 0.47%; *p* = 0.009) (Figure 4B). In both cohorts, CD11B^+^ cells increased at day 1 over baseline levels (Figure 4C) (cohort 1, 93.09 ± 3.70 vs. 91.53 ± 3.43%, *p* = 0.044; cohort 2, 94.60 ± 8.70 vs. 92.01 ± 3.63%, *p* = 0.005); the percentage of CD11B+ cells was significantly higher over baseline at day 30 only in cohort 2 (93.92 ± 1.91 vs. 92.01 ± 3.63%, *p* = 0.020). In cohort 1, progenitor cells (CD45^+^CD133^+^) decreased significantly at day 30 (0.724± 0.12%) over baseline levels (1.303 ± 0.24%; *p* = 0.016) (Figure 4D). We did not find any statistical differences between the two cohorts in the other cell markers analyzed in this study.

### Baseline B Cells Predict Positive Clinical Outcome

**Figure 5:**
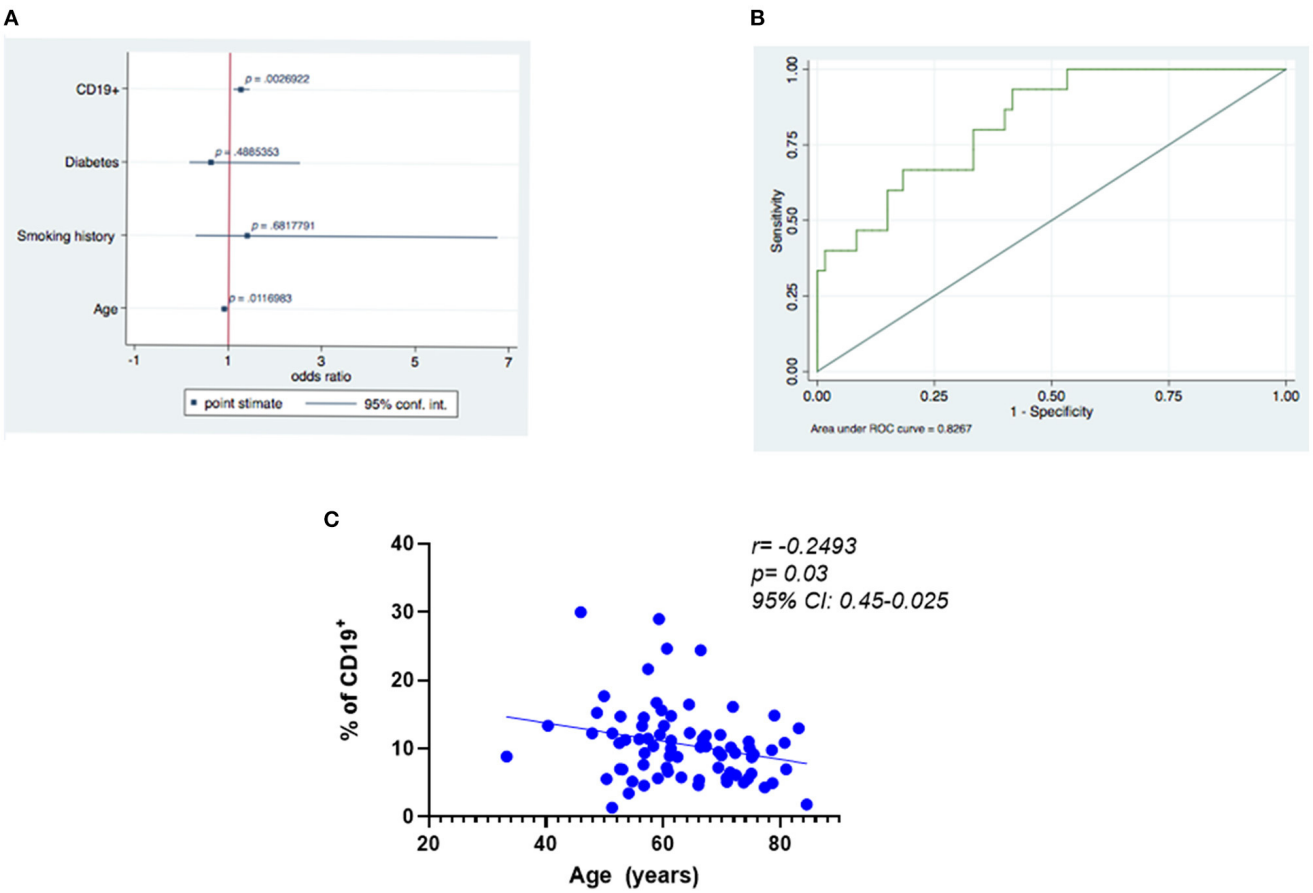
Predictors of best functional outcome using CD19^+^ cells at baseline. **(A)** Outcome variable: best functional outcome (increase in LVEF and VO_2_ max and decrease in LVESV), multivariate analysis: logistic regression (significance of the model *p* ≤ 0.001). **(B)** Receiver operating characteristic (ROC) curves relating to the ability of baseline CD19^+^ cells to predict improvement in the three functional outcomes was 0.83. **(C)** Negative correlation between age and the percentage of positive CD19^+^.

Logistic regression analysis demonstrated that the likelihood of a positive outcome was associated with the percentage of CD19+ B cells at baseline (Figure 5A; odds ratio [OR], 1.26; 95% confidence interval [CI] = 1.083–1.462; *p* = 0.003) and was negatively related to age at baseline (OR = 0.89; 95% CI = 0.817–0.963; *p* = 0.004) (Figure 5B; receiver operating characteristic curve, 0.83). Additionally, we found a negative correlation between age and the percentage of positive CD19+ cells (Figure 5C; *r* = −0.25, *p* = 0.02493, 95% CI: 0.45–0.025). No correlation was found between CD19+ cells, diabetes (*r*= −0.054, *p* = 0.65, 95% CI 0.2826–0.1798), and history of smoking (*r* = −0.061, *p* = 0.60; 95% CI 0.2888–0.1733).

## Discussion

In the current analysis, we identified circulating cell populations (CD45^+^CD19^+^, CD19^+^CD11B^+^, CD11B^+^, and CD45^+^ CD133^+^ cells) that were associated with three improved functional cardiac outcomes (LVEF, LVESV, and VO_2_ max) in patients with chronic HF in the FOCUS-CCTRN trial. To our knowledge, this is the first study to demonstrate an association between circulating levels of B cells at multiple time points with improved LV function in patients with HF of ischemic etiology. Specifically, we showed that patients with best functional outcomes (cohort 1) had a higher percentage of B cells (CD45^+^CD19^+^) at days 0 (baseline), 1, 90, and 180 than did patients with worst functional outcomes (cohort 2). Our results suggest that B cells may play a protective role in chronic LV dysfunction HF. Our novel findings may help in developing biomarkers for selecting patients who are more likely to respond in clinical studies of cell therapy. Moreover, our observations are relevant for understanding the role of immune and reparative responses in the progression of HF at the cellular level.

Although data on the role of B cells in the natural progression of HF are limited, some B-cell dependent mechanisms have been described in cardiovascular diseases (17). Molina et al. demonstrated that B cell (CD19^+^) lymphopenia at baseline may be an independent predictor of higher cardiovascular mortality at 1 year in hemodialysis patients (18). In a *post-hoc* analysis of the EVEREST trial (NCT00071331), Vaduganathan et al. found that low lymphocyte counts in blood from patients hospitalized for HF can independently predict poor outcomes after discharge (19). An et al. showed that advanced donor age impairs the therapeutic potential of bone marrow cells by reducing B cell levels in patients after myocardial infarction (20). Consistent with this observation, patients in cohort 2 were significantly older than those in cohort 1.

In cardiac biopsy specimens from patients with end-stage HF due to ischemic and non-ischemic cardiomyopathy, Youker et al. showed that IgG antibodies were present in 71% of specimens (21). In a mouse model of angiotensin-II-induced HF, B cells played a role in the production of tissue-specific antibodies that contributed to cardiac injury (22). Moreover, in animal models, B lymphocytes have been reported as a therapeutic bone marrow subpopulation (14, 23) and as protective against ischemic brain injury (5). In a mouse model, decreased B cell levels were associated with worsening of atherosclerosis after splenectomy (24). Indeed, B cells (CD19^+^) appear to have a role in HF. However, the molecular process in myocardial biology remains a relatively unexplored area.

Levels of the B-cell subset CD19^+^CD11B^+^ were significantly higher at baseline and at day 90 in cohort 1 than in cohort 2, but they were higher at day 1 and significantly increased at day 30 in cohort 2. Kawai et al. reported that the CD11B antigen contributes to the migratory ability of PB-derived B cells and is highly expressed in memory cells (25), suggesting that this antigen plays a central role in the infiltration of B cells into tissues.

We also observed an increase in monocyte-derived cells that were double-positive for CD45^+^ and CD11B^+^ (Figure 4C). In both cohorts, the percentage of cells that expressed CD11B^+^, an integrin found on monocytes, neutrophils, lymphocytes, and natural killer cells, increased at day 1 over baseline levels. This finding may suggest a response to the bone marrow aspiration performed the day before or a response to the injection of the BM-MNCs or placebo. Interestingly, only in cohort 2 did the increase persist until day 30, which may suggest an extended activity period for inflammatory monocytes. A controlled recruitment of monocytes is critical for host defense, wound healing, and tissue repair; however, a disproportionate and prolonged presence of inflammatory monocytes is a hallmark of diseases such as HF (26) that have an inflammatory component, and this persistence of monocytes may lead to cardiac tissue damage and inflammatory cytokine release (27). In chronic HF, inhibiting the recruitment of CD11B+ cells from the spleen to the heart prevents inflammation and remodeling of cardiac tissue (28). More studies are needed to fully understand the role of CD11B+ cells in HF and to identify the mechanisms necessary to achieve a balance in the contrasting roles of monocytes/macrophages (10, 29).

At day 30, cohort 1 had decreased levels of CD45^+^CD133^+^ cells as compared with cohort 2 (Figure 4D). The functions of the cell surface antigens CD45 and CD133 are unknown, but they are expressed on early progenitor cells (30–33). Among stem cell markers, CD133 is thought to denote one of the most immature stem cell populations. Previous clinical work has demonstrated a central regulatory role of CD133^+^ cells in angiogenesis (34). In preclinical models, CD133^+^ cells provided benefits in angiogenesis and improved myocardial contractility (35). In post-infarction patients with chronic ischemia and reduced LVEF, Steinhoff et al. demonstrated that an induction of cardiac repair correlated with CD133^+^ cells released from the bone marrow (34). Using cardiac magnetic resonance imaging, Forcillo et al. demonstrated an increase in systolic wall thickening and mean segmental wall thickness at 18 months after intramyocardial injection of CD133^+^ cells in patients with chronic ischemic cardiomyopathy (36). Our findings suggest the circulating progenitor CD133^+^ cells may have migrated into cardiac tissue and promoted a reparative effect in cohort 1. Whereas our group previously reported significant differences in CD45^+^CD3^bright^, CD19^+^CXCR4^+^ cells in the bone marrow of the study population (7), we did not observe any differences in PB, suggesting that the cells either had already trafficked to the tissues or had not yet mobilized into the periphery.

Although we found no differences in the three primary endpoints for cardiac function (LVEF, LVESV, and VO_2_ max) at baseline between the two cohorts, baseline BNP levels were significantly lower in cohort 1. Brain natriuretic peptide is secreted by ventricular myocytes in response to wall tension, stress, and LV stretching (37) and is associated with the progression and severity of HF (38–40). Interestingly, the percentage of patients with NYHA class III HF symptoms was 23.53% in cohort 1 and 63.64% in cohort 2. Thus, despite there being no differences in the three endpoints between the two groups, patients in cohort 1 were younger and had fewer physical limitations and better functional status at baseline.

Our study has limitations. To better characterize the relationship between changes in PB cell subsets and cardiac function, parameters such as LVEF, VO_2_ max, and LVESV should be evaluated at earlier time points (i.e., 30 and 90 days). However, those data were not available.

## Conclusions

We have identified a set of circulating cell populations associated with improved cardiac function in patients with ischemic HF. Our findings may help identify biological variables that could potentially contribute to the selection of patients with a beneficial phenotypic profile for cell therapy trials and, thereby, enable more targeted precision therapy.

## Data Availability Statement

The raw data supporting the conclusions of this article will be made available by the authors, without undue reservation.

## Ethics Statement

The studies involving human participants were reviewed and approved by the FOCUS trial and were monitored by the NHLBI-sponsored Gene and Cell Therapy Data and Safety Monitoring Board. The BRC protocol was approved by the committee for the protection of human subjects at the University of Texas Health Science Center at Houston. Written informed consent for participation was not required for this study in accordance with the national legislation and the institutional requirements.

## Author Contributions

LCA: conception and design, assembly of data, data analysis and interpretation, manuscript writing, and final approval of the manuscript. EP, JW, RB, PY, JT, DL, and CP: conception and design, administrative support, provision of study patients, collection of the data, data interpretation, manuscript writing, and final approval of the manuscript. AG and DT: conception and design, administrative support, collection of the data, data analysis and interpretation, manuscript writing, and final approval of the manuscript. All authors contributed to the article and approved the submitted version.

## Author Disclaimer

The opinions expressed in this report do not necessarily reflect those of the National Heart, Lung, and Blood Institute, the NIH, or the US Department of Health and Human Services.

## Conflict of Interest

DT has a financial interest in Miromatrix Medical, Inc. DT have financial interests in Stem Cell Security, LLC. This relationship was monitored in accordance with the conflict of interest policies by the Texas Heart Institute. The remaining authors declare that the research was conducted in the absence of any commercial or financial relationships that could be construed as a potential conflict of interest.

## Publisher's Note

All claims expressed in this article are solely those of the authors and do not necessarily represent those of their affiliated organizations, or those of the publisher, the editors and the reviewers. Any product that may be evaluated in this article, or claim that may be made by its manufacturer, is not guaranteed or endorsed by the publisher.
